# Around the Hospitals

**Published:** 1893-10-07

**Authors:** 


### Around the Hospitals.

Notice to the Managers of Institutions.—Special space is now reserved for the insertion of notices of hospital meetings and festivals. The Editor requests that all notices may reach The Hospital Office, 428, Strand, at least one week before the date of each meeting.

**The Worthing Infirmary.**—Owing to the epidemic of typhoid at Worthing the infirmary has had severe stress laid upon it. In order to cope with the exigencies of the case tents were erected for the reception of patients in the infirmary grounds, besides setting aside beds for the treatment of typhoid cases. The Town Council cases repays the expenditure attendant on the care of the sick received in the tents, but the epidemic has caused unusual expenses to the institution, for which it will be difficult to obtain extra contributions, as the wide-spread distress caused by the visitation in the town must naturally direct money into other channels. The annual report of the institution testifies to the excellence of the services of Miss Jury, the Matron.

**The Sheffield General Infirmary.**—The work of the Sheffield Infirmary is increasing. During the past year 1,643 in-patients and 3,565 out-patients have been treated, and it is found most necessary to establish a new department for the treatment of skin diseases. Yet, if either the present work is to be uncurtailed, much less a fresh field entered on, the well-to-do in Sheffield must respond more generously to its necessary and moderate demands. The institution showed a deficiency of £1,808 on last year's accounts. The whole sum received during the same period amounted to £1,175. Of this the local Hospital Sunday Fund contributed £694, and, as we are informed that the industrial classes are hearty supporters of their hospital, what a very small sum remains as the contribution of the wealthier. We do not overlook the facts that this class contributes largely to the Sunday fund, but such contribution should be held as altogether outside the householders' annual subscription, which it should be regarded as a privilege and duty of all good citizens to render to an institution which benefits both rich and poor. It has always been found that care rendered to children excites a large amount of interest, and as the children's department is an unusually large one, it should help to secure funds for the whole institution. The infirmary, originally situated half a mile from the town, now is surrounded by habitations on all sides, a fact that renders the work of those in charge of the sick to be more difficult than before. It is pleasing to learn that the services of Mr. Favell, which have covered a period of twenty-five years, are warmly appreciated, and on his retirement from the medical staff of the infirmary, which is now announced, he has been appointed honorary consulting surgeon, and three beds have been allotted to him for the reception of cases to be treated by him.

